# Practitioners, the Hospitals, and the Poor Law

**Published:** 1911-04-22

**Authors:** 


### The Hospital

A Journal of

The Medical Sciences and Hospital Administration.

New Series. No. 220, Vol. IX. [No. 1292, Vol. L.] SATURDAY, APRIL 22, 1911

### PRACTITIONERS, THE HOSPITALS, AND THE POOR LAW.

During the last fifty years, owing to the rapid development of modern medical and surgical methods which demand the use of complicated and expensive apparatus, a well-organised hospital has become a *sine qua non* in every community. As a result of this the general practitioner has been forced to help in the establishment of local hospitals, notwithstanding the fact that he is acutely conscious of the detriment that his practice suffers through the competition with such institutions. It is therefore not to be wondered at that there is a tendency on his part to dilate on the demerits of a system which allows such unrestricted competition, and thereby adds to the already severe struggle which the practitioner—and especially the young practitioner who is starting his career—has to face in a locality where a large hospital with an unrestricted out-patient department exists. On the other hand, it is only fair to add that many practitioners are cordially in favour of hospitals so long as these institutions confine their work to the poorer sections of the community whose return for medical treatment is utterly inadequate when the amount of time and care spent on their cases by the practitioner is considered.

Let us take as an instance the case of a middle-class practitioner with a comparatively large club service. A club patient attends him with an injury to the shoulder-joint which needs not only careful treatment, but—a matter of much greater importance—exceedingly careful diagnosis and examination. Such examination demands time, patience, practice in similar cases, wide experience, and careful judgment. Admitting—which is not always a fact—that the practitioner possesses all these, there yet remains the final examination under the $x$-rays, which nowadays, in view of the decisions by courts of law in cases of alleged negligence, no practitioner can afford to neglect in the poorest patient. The average practitioner, however, is not in a position to keep and work a proper $x$-ray apparatus, nor is he, in the vast majority of cases, in a position to judge the results of such examination. In the case of a well-to-do patient he can easily obtain expert aid; he can send his patient to a private $x$-ray specialist and obtain both a photograph and an opinion on it—a procedure which involves no expense to him, since the patient pays for it, and by which he incurs no risk, since the specialist is not likely to "steal" his patient. With the poor he cannot afford to do so, since the expenses must fall upon himself. At the same time he cannot, in his own interests, afford to neglect the examination, since even a club patient may be awarded heavy damages for the results which follow a line of treatment based on a false diagnosis. In this emergency the hospital comes to his aid. It undertakes examination and treatment, while at the same time it enables him to follow the results of such treatment. We may instance other examples—cases in which light or radium treatment is required, in which major operations are to be performed, or in which inoculations are prescribed. None of these cases, especially when the patients are poor, can be efficiently treated at home; they are not, it is true, necessarily in-patients, but they form a class which must be attended to under conditions such as obtain only in a properly equipped, up-to-date, modern hospital.

The general practitioner, we imagine, would be the last person in the world to deny the value of the voluntary hospital, as opposed to the local infirmary, in the treatment of such cases. Apart altogether from the presupposition—whether groundless or not we need not now discuss—that attendance at an infirmary entails a stigma of pauperism, it is generally accepted that special, or what may be called departmental, treatment is better carried out at a voluntary hospital than at an infirmary. Add to this the fact that the hospital staff is usually well known to the practitioner, that its members are his personal friends, teachers, or old fellow-students, and it will readily be conceived that the preference which the practitioner in general gives to the voluntary hospital when an opinion is required or a special line of treatment has to be carried out, is reasonable in the circumstances. But with all that it is useless to blink the fact that the practitioner can draw up a heavy case against the voluntary hospital from his own experience. Into the separate items of that indictment we need not enter now, though we hope shortly to publish articles from the pens of well-known general practitioners elaborating this side of the question. We are for the moment merely concerned with the fact that if there is much to be said in favour of the hospital from the practitioner's point of view, there is almost equally much to be said against it.

The reason for the existence of these drawbacks to a system which is on the whole so well suited to medical practice in this country are to be found in two things mainly. These are the unrestricted extension of the work in the out-patient department, and the want of differentiation between different classes of patients who now attend hospitals. In other words, the root of the trouble lies in the absence of co-operation between the general practitioner and the hospitals. When such co-operation has been secured and when it has been established on a firm basis after mutual discussion, these difficulties and objections will almost automatically abolish themselves. The establishment of a proper system of supervision by means of experienced almoners, and the abolition of the present out-patient department with the substitution for it of a reorganised casualty and consultation departments somewhat after the manner of the cliniques now held at the Polyclinic, appear to us to be essential preliminaries to such co-operation. It is unreasonable to expect the private practitioner to look calmly on any hospital which exploits his practice, by taking into its wards patients who are able to pay for private treatment; it is equally unreasonable to demand that he shall establish in his own consulting room a miniature hospital on the most up-to-date model and replete with all the necessary apparatus for special work. What is wanted is a friendly system of selection; a weeding out, on the part of the institutions, of those patients who come to them for free medical relief while in a position to pay for such relief, and a similar selection on the part of the medical man of such patients as he cannot treat adequately with the means at his command, but who can be relieved at a hospital. That is the principle of co-operation between hospital and practitioner which we should like to see established; for we feel sure, when once it has been acknowledged and carried into effect, the competition which the practitioner now finds so hard and so justifiably inexcusable will cease to exist, and with it the objection to the voluntary system which some medical men still cling to.

